# Mesenteric Fat Necrosis Mimicking a Malignant Mesenteric Tumor: A Case Report

**DOI:** 10.70352/scrj.cr.26-0024

**Published:** 2026-05-26

**Authors:** Aoki Uchikoshi, Yoshihisa Okuchi, Koji Hisano, Meiki Fukuda, Wataru Fukunaga, Takaaki Yakushigawa, Yuki Kawai, Yoshiki Oshimo, Kenzo Nakano, Takayuki Kawai, Hisatsugu Maekawa, Kohta Iguchi, Eiji Tanaka, Kojiro Taura, Hiroaki Terajima

**Affiliations:** Department of Gastroenterological Surgery and Oncology, Kitano Hospital Medical Research Institute, 2-4-20 Ogimachi, Kita-ku, Osaka, Osaka 530-8480, Japan

**Keywords:** mesenteric fat necrosis, sclerosing mesenteritis, mesenteric mass, laparoscopic resection, case report

## Abstract

**INTRODUCTION:**

Mesenteric fat necrosis, an uncommon benign condition, can present as a mass-forming lesion and can closely mimic malignant mesenteric disease on imaging. Because its radiologic findings may overlap with those of malignant mesenteric tumors, preoperative diagnosis can be challenging, particularly when tissue sampling is difficult. This report describes a rare case of mesenteric fat necrosis, presenting as an enlarging mesenteric mass suspicious for malignancy.

**CASE PRESENTATION:**

An 85-year-old man who had undergone laparoscopic low anterior resection for rectal cancer 13 years prior was found on follow-up imaging to have an enlarged mass at the jejunal mesenteric root. The lesion showed contrast enhancement on CT, diffusion restriction on MRI, and increased ^18^F-fluorodeoxyglucose uptake on PET, strongly implying a malignant mesenteric tumor, including primary or metastatic mesenteric neoplasms. The biopsy was not feasible because of its deep mesenteric location. Therefore, laparoscopic partial resection of the jejunum containing the mass was performed for definitive diagnosis and treatment. Intraoperatively, a well-circumscribed mass was identified at the mesenteric root of the jejunum. Approximately 30 cm of jejunum was resected. Histopathological examination revealed fibrosis and fat necrosis without malignant features. He recovered uneventfully. No recurrence was observed during 1 year of follow-up.

**CONCLUSIONS:**

Mesenteric fat necrosis can closely mimic malignant mesenteric tumors on multimodality imaging and should be considered in the differential diagnosis of an enlarging mesenteric mass. When biopsy is not feasible, but imaging findings raise concern about malignancy, surgical resection can be a definitive diagnostic and therapeutic option.

## Abbreviations

ADC
: apparent diffusion coefficient
CA19-9
: carbohydrate antigen 19-9
CEA
: carcinoembryonic antigen
CE-CT
: contrast-enhanced CT
CE-MRI
: contrast-enhanced MRI
DWI
: diffusion-weighted imaging
FDG-PET
: ^18^F-fluorodeoxyglucose PET
GIST
: gastrointestinal stromal tumor
T1WI
: T1-weighted images
T2WI
: T2-weighted images

## INTRODUCTION

Mesenteric fat necrosis, an uncommon benign inflammatory condition, might present as a mass-like lesion in the mesentery and might closely mimic malignant disease on cross-sectional imaging.^1–5)^ Adipose tissue is a metabolically active tissue. When it undergoes ischemic changes related to various causes, it might occasionally give rise to tumor-like fat necrosis.^6–8)^ Historically, mesenteric fat necrosis has been regarded as part of the spectrum of sclerosing mesenteritis, a disorder characterized by varying degrees of fat necrosis, chronic inflammation, and fibrosis.^4,5,7,9,10)^ Although many cases within this spectrum are detected incidentally or show diffuse mesenteric involvement, isolated mass-forming mesenteric fat necrosis appears to be uncommon.^1,3,6,11,12)^

Because its radiological features, including contrast enhancement or ^18^F-fluorodeoxyglucose uptake, often overlap with those of malignant tumors, distinguishing it from neoplastic processes can be challenging. The differential diagnosis of a solid mesenteric mass includes lymphoma, desmoid-type fibromatosis, liposarcoma, metastatic disease, and other mesenteric neoplasms.^1,3,4,6,8,12,13)^

Prior abdominal surgery, trauma, ischemia, and other inflammatory insults have been suggested as one of several possible contributing factors, but their pathogenesis remains unclear.^3,4,7,9–12)^

Herein, we present a case of fat necrosis arising within the small intestinal mesentery in a patient with prior rectal cancer surgery. The lesion presented as an enlarging deep mesenteric mass with multimodality imaging findings suspicious for malignancy and ultimately required surgical resection for definitive diagnosis.

## CASE PRESENTATION

**Fig. 1:**
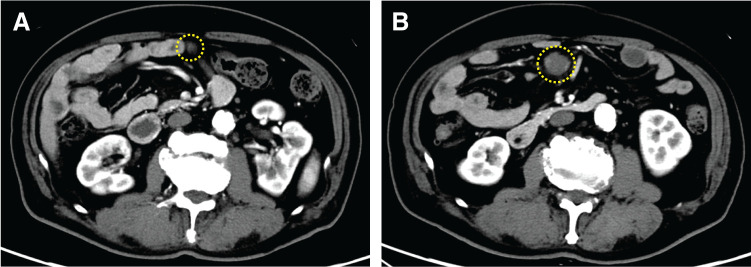
Abdominal CE-CT images taken 14 months prior (**A**) and at initial consultation (**B**). The mass lesion in the small intestinal mesentery showed contrast enhancement; it had become larger during this period (yellow circle outlines). CE-CT, contrast-enhanced-CT

An 85-year-old man (BMI, 26.6 kg/m^2^) had undergone laparoscopic low anterior resection for rectal cancer (pT3N1M0; pStage IIIa, according to the *Japanese classification of Colorectal Appendiceal, and Anal Carcinoma*, *Seventh Edition*) 13 years prior. He had been under follow-up for suspected hepatocellular carcinoma detected 9 years prior. His past medical history included appendectomy, oral cancer, diabetes, and Hashimoto’s disease. Follow-up CE-CT revealed an approximately 2-cm mass in the small intestinal mesentery, which showed gradual enlargement and contrast enhancement. During 14 months, the lesion size increased from 13 × 8 to 21 × 20 mm, as observed retrospectively (**Fig. 1A** and **1B**). Serum tumor markers, including CEA and CA19-9, remained within the normal limits. Because follow-up imaging demonstrated an enlarging mesenteric mass with suspicious features, the patient was referred to our department for additional diagnostic evaluation.

**Fig. 2:**
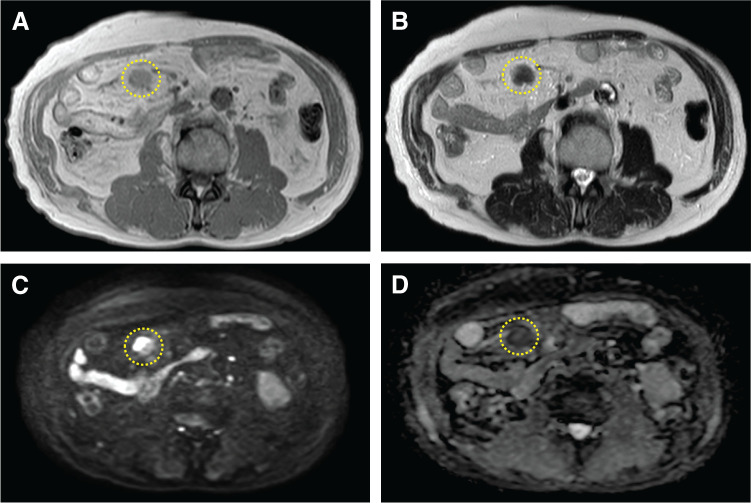
CE-MRI images show a mesenteric mass (yellow circle outlines) with slightly higher signal intensity than muscle on T1WI (**A**) and with an isointense signal on T2WI (**B**). The lesion shows diffusion restriction on DWI (**C**) and reduced ADC values on the ADC map (**D**). ADC, apparent diffusion coefficient; CE-MRI, contrast-enhanced-MRI; DWI, diffusion-weighted imaging; T1WI, T1-weighted images; T2WI, T1-weighted images

**Fig. 3:**
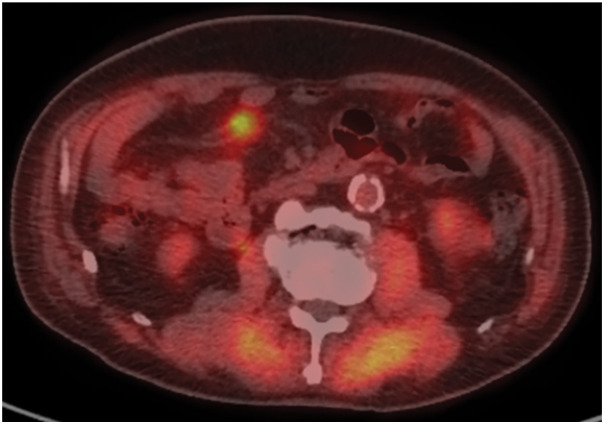
FDG-PET reveals focal uptake in the same region corresponding to the mass observed on CT and MRI. FDG-PET, ^18^F-fluorodeoxyglucose-PET

At the time of his visit to our hospital, he was asymptomatic. Routine laboratory test results revealed no marked abnormality. He was taking medications for his preexisting conditions. CE-MRI showed a mesenteric mass with slightly higher signal intensity than muscle on T1WI and with an isointense signal on T2WI (**Fig. 2A** and **2B**). The lesion showed diffusion restriction on DWI and reduced ADC values on the ADC map (**Fig. 2C** and **2D**). Furthermore, FDG-PET revealed focal uptake corresponding to the same lesion identified using CT and MRI (**Fig. 3**). Considering the possibility of new malignant tumors other than rectal cancer, upper and lower endoscopies were performed, but no malignant findings were observed.

**Fig. 4:**
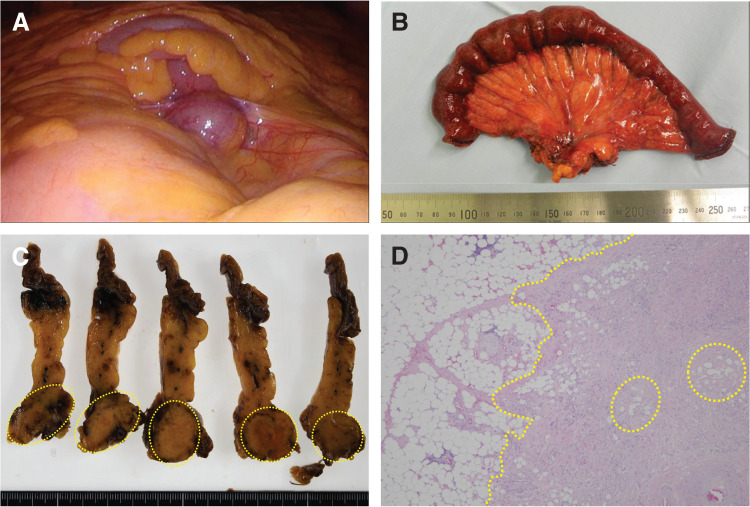
Intraoperative and pathological findings. (**A**) Laparoscopic view of the abdominal cavity shows a mass identified in the mesentery of the jejunum. (**B**) Approximately 30 cm of the jejunum containing the mass is resected. (**C**) Cross-sectional appearance of the specimen. The mass was located at the base of the resected specimen (yellow circle outlines). (**D**) HE staining shows normal adipose tissue and fibrotically thickened adipose tissue containing scattered foci of necrotic fat (yellow circle outlines), with a yellow dotted line representing their border. HE, hematoxylin and eosin

Based on the imaging findings, a malignant mesenteric disease could not be ruled out. The differential diagnosis included lymphoma, desmoid-type fibromatosis, liposarcoma, GIST, and late recurrence of rectal cancer. Because the lesion was located deep within the mesentery and because it was challenging to access endoscopically for biopsy, surgical resection was considered necessary for both diagnostic and therapeutic purposes after obtaining informed consent. Laparoscopic exploration identified the mass at the mesenteric root of the jejunum (**Fig. 4A**). Because the mass involved the mesenteric root, several small intestinal branches arising from the superior mesenteric artery were encased by the mass and required ligation, thereby rendering limited small-bowel resection impractical. Consequently, approximately 30 cm of the jejunum containing the mass was resected (**Fig. 4B**). Histopathological examination revealed normal adipose tissue with increased fibrosis and fat necrosis. Nevertheless, no evidence of malignancy was observed (**Fig. 4C** and **4D**). These findings were consistent with mesenteric fat necrosis within the spectrum of sclerosing mesenteritis. The patient progressed favorably after the operation. He was discharged on the eighth postoperative day. One year after the surgery, he has shown no recurrence.

## DISCUSSION

This report describes a case of mesenteric fat necrosis, an uncommon non-neoplastic inflammatory disease that can closely mimic malignant mesenteric tumors. This case highlights the diagnostic challenge posed by mesenteric fat necrosis, particularly when it presents as an enlarging mass with contrast enhancement, diffusion restriction, and FDG uptake, which strongly suggest malignancy on cross-sectional imaging, while a safe preoperative biopsy is not feasible. In such circumstances, mesenteric fat necrosis should be considered in the differential diagnosis; however, surgical resection was ultimately necessary to produce a definitive diagnosis and treatment when malignancy could not be confidently excluded.

Historically, lesions in the mesentery associated with varying degrees of fat necrosis, chronic inflammation, and fibrosis have been described as distinct entities: mesenteric lipodystrophy, mesenteric panniculitis, and retractile mesenteritis.^14–16)^ These conditions are now understood to represent histologic variants along a continuous spectrum, designated collectively as sclerosing mesenteritis.^3,7,14)^ Within this spectrum, lesions dominated by fibrosis and fat necrosis may present as localized mass-forming lesions and can therefore closely resemble mesenteric neoplasms. Although its etiology remains unclear, several factors such as trauma, infection, ischemia, prior abdominal surgery, and autoimmune or inflammatory mechanisms have been suggested as potential factors.^3,7,17)^ However, these associations are nonspecific, and a direct causal relationship is often difficult to establish in an individual case. In the present case, previous abdominal surgery may have represented a background factor, but the lesion should not be interpreted as specifically related to prior rectal cancer surgery.^9,14)^

Clinical presentation of lesions within the spectrum of sclerosing mesenteritis varies widely. Many patients present initially with nonspecific gastrointestinal symptoms such as abdominal pain, nausea, vomiting, or altered bowel habits. By contrast, as in the present case, some cases remain completely asymptomatic. Also, lesions are detected incidentally during imaging studies.^4,7,15,16,18)^ The imaging characteristics of mesenteric fat necrosis are also heterogeneous. In this case, the lesion showed interval growth, contrast enhancement on CE-CT, high signal intensity on T1WI compared with muscle, an isointense signal on T2WI, diffusion restriction on DWI, and increased FDG uptake. These features were atypical for a confidently benign lesion and contributed to the diagnostic difficulty. The degree of enhancement and signal intensity can differ considerably depending on the proportions of necrosis, inflammation, and fibrosis. Some cases have shown no contrast enhancement on CE-CT,^1)^ but others have exhibited isointensity on T1WI and predominantly low signal on T2WI.^6)^ These variations among findings highlight the difficulty of producing a diagnosis based solely on preoperative imaging.

Given this variation, the differential diagnosis of an enlarging solid mesenteric mass is broad, ranging from benign inflammatory conditions to malignant tumors such as lymphoma, GIST, liposarcoma or other mesenteric sarcoma, desmoid-type fibromatosis, or metastatic mesenteric disease.^8,13)^ These differential diagnoses exhibit characteristic imaging findings (**Table 1**)^7,8,13,19–27)^; however, in general, most solid mesenteric masses are neoplastic, with sclerosing mesenteritis representing an important benign exception.^8)^ Therefore, when a lesion demonstrates interval growth and malignant-appearing multimodality imaging findings, histopathological evaluation is often indispensable.

**Table 1:** Differential diagnosis of an enlarging mesenteric mass in the present case and its radiologic features

Disease	Typical imaging clues	Findings in the present case
Lymphoma^20,21)^	CT: Bulky homogeneous soft-tissue mass or confluent nodal mass; mesenteric vessels may be engulfed without marked narrowing (“sandwich sign”). MRI: Usually relatively homogeneous soft-tissue signal. PET: Often FDG-avid.	CT: Deep mesenteric-root mass with interval growth was compatible. MRI: Diffusion restriction could support lymphoma. PET: Focal FDG uptake supported consideration. Against: No clear bulky nodal conglomerate or classic sandwich sign was described; lesion was relatively small and well-circumscribed.
GIST^8,22)^	CT: Well-defined enhancing mass, often exophytic from bowel; necrosis or hemorrhage may be present. MRI: Variable, often heterogeneous with enhancement. PET: Can be FDG-avid.	CT: Enhancing lesion adjacent to small bowel mesentery made GIST/EGIST a consideration. MRI: Diffusion restriction did not exclude it. PET: FDG uptake was compatible. Against: No definite bowel-wall origin or exophytic intestinal mass was identified radiologically or intraoperatively.
Liposarcoma/other mesenteric sarcoma^13,23)^	CT: Fatty mass with thick septa, nodular non-fatty soft-tissue components, or marked heterogeneity; non-lipomatous sarcomas appear as solid malignant mesenteric masses. MRI: Fat-containing lesions are characterized well; nodular soft-tissue components increase concern. PET: Variable, often higher in more aggressive non-fatty components.	CT/MRI: A malignant mesenteric sarcoma remained a reasonable broad differential because of interval growth and enhancement. PET: FDG uptake supported concern for malignancy. Against: The lesion was not described as a predominantly fatty tumor with thick septa or nodular soft-tissue elements typical of liposarcoma.
Desmoid-type fibromatosis/mesenteric fibromatosis^24,25)^	CT: Solid soft-tissue mesenteric mass, often well-defined but locally infiltrative; may encase bowel or mesenteric vessels. MRI: Variable signal; often low to intermediate T1 and heterogeneous T2 depending on collagen/cellularity; enhancement is variable. PET: Uptake is variable and usually not highly specific.	CT: Deep mesenteric location and progressive growth were compatible. MRI: Fibrous tumor remained plausible despite nonspecific signal. PET: Uptake did not exclude desmoid but increased concern for malignancy. Surgical correlation: Involvement of small intestinal arterial branches made this a realistic preoperative consideration.
Mesenteric carcinoid/small-bowel NET metastasis^13,26,27)^	CT: Spiculated mesenteric mass with desmoplastic reaction; calcification may be present. MRI: Fibrotic/desmoplastic components may show relatively low T2 signal. PET: FDG uptake is variable; somatostatin receptor imaging is often more informative, but malignant-appearing uptake can occur.	CT: Mesenteric-root location was compatible with a mesenteric metastatic deposit. MRI: Some fibrotic components could fit. PET: Focal uptake supported a neoplastic differential. Against: No calcification, no obvious radiating desmoplastic strands, and no definite small-bowel primary lesion were identified.
Metastatic mesenteric disease/carcinomatosis (including late colorectal cancer recurrence)^13)^	CT: Enhancing mesenteric nodules or masses, sometimes multiple; interval growth favors malignancy. MRI: Variable, depending on tumor type. PET: May show focal uptake if metabolically active.	CT: Prior rectal cancer history, solitary enlarging enhancing mesenteric lesion supported metastatic disease as a practical concern. MRI: Diffusion restriction was compatible. PET: FDG uptake increased suspicion. Against: Solitary lesion, jejunal mesenteric-root location, and very long postoperative interval made rectal cancer recurrence less convincing as the leading diagnosis.
Mass-forming sclerosing mesenteritis/ mesenteric fat necrosis^7,8)^	CT: Variable; may appear as a localized mesenteric mass rather than classic diffuse “misty mesentery.” MRI: Signal depends on proportions of fat necrosis, inflammation, and fibrosis; no single feature is diagnostic. PET: Often non-avid, but focal uptake can occur and may mimic malignancy.	CT: Localized mesenteric lesion was compatible, but interval growth and contrast enhancement were worrisome. MRI: The lesion was not confidently benign because it showed diffusion restriction with low ADC. PET: Focal FDG uptake strongly mimicked malignancy. Pathology: Fibrosis and fat necrosis ultimately established this diagnosis.

ADC, apparent diffusion coefficient; EGIST, extra-gastrointestinal stromal tumor; FDG, ^18^F-fluorodeoxyglucose; GIST, gastrointestinal stromal tumor; NET, neuroendocrine tumor

In the present case, increased FDG uptake on PET further complicated the preoperative assessment. Although inflammatory mesenteric lesions are often non-avid on FDG-PET, focal FDG uptake can occur and may closely mimic malignant involvement, especially in patients with a history of cancer.^28,29)^ Therefore, FDG avidity should not be interpreted as pathognomonic for malignancy; however, when combined with the findings above, it substantially increased concern for a malignant mesenteric lesion, supporting the decision to pursue operative management in the present case.

To contextualize the present case, we performed a structured literature search of PubMed from database inception to February 2026 using the terms “mesenteric fat necrosis,” “encapsulated fat necrosis,” “mesenteric lipodystrophy,” “sclerosing mesenteritis,” “mesenteric panniculitis,” and “retractile mesenteritis.” The initial search yielded 774 records, of which 541 had abstracts available. After title and abstract screening, 75 case reports referring to fat necrosis were identified for further review. Reports describing encapsulated fat necrosis of the skin or extremities or an isolated mass in the abdominal cavity were excluded. Because pathologically confirmed mesenteric fat necrosis was extremely uncommon, we also included adult mass-forming cases diagnosed as sclerosing mesenteritis, mesenteric panniculitis, or retractile mesenteritis when they were clinically relevant to the differential diagnosis of malignant mesenteric tumors. Restricting the review to English-language reports with accessible full text yielded 31 reports for detailed assessment, from which clinically comparable cases were selected for structured comparison in **Table 2**.^1,6,11,30–57)^

**Table 2:** Previously reported cases of mesenteric fat necrosis and mass-forming sclerosing mesenteritis

	PMID	Year	Age	Sex	Chief complaint	Past history	Urgent complications	Imaging findings	Biopsy	Diagnosis	IgG4-related	Surgical intervention	Size of the mass (cm)	Medications	Prognosis
1	3308370	1987^30)^	57	Male	A change in bowel habits	None	None	—	—	MP	—	Complete resection	10 × 15	None	No recurrence
			61	Male	Abdominal pain	None	None	CT: An ill-defined mass of the sigmoid mesentery infiltrating the distal descending and sigmoid region	—	MP	—	Complete resection	—	None	No recurrence
2	8782303	1996^31)^	46	Female	Constipation	Appendectomy	None	CT: An irregularly shaped mass with a density consistent with that of soft tissue. MRI: An irregularly shaped mass growing around the inferior mesenteric artery that demonstrated intermediate-intensity signals consistent with those of soft tissue on both T1WI and T2WI. US: An irregularly shaped, ill-defined hypoechoic mass	—	RM	—	Complete resection	10 × 5 × 5	None	No recurrence
3	15091122	2004^32)^	55	Male	Abdominal pain, distention	Appendectomy	None	CT: A well-circumscribed intraperitoneal mass of fat density with an enhanced thick wall with calcification	—	Encapsulated FN	—	Complete resection	7 × 3 × 8	None	—
4	17910751	2007^33)^	63	Male	Abdominal pain, weight loss, constipation	None	None	CT: Negative except for a small amount of ascites	—	MP	—	Complete resection	—	None	No recurrence
5	18925952	2008^34)^	52	Male	Abdominal pain, intra-abdominal mass	None	None	CT: Solid soft-tissue mass with calcification	Surgical biopsy	SM	—	Complete resection	Approximately 5	None	No recurrence
6	19091063	2008^35)^	62	Male	Abdominal pain, fatigue, weight loss, nausea, vomiting	Non-Hodgkin lymphoma	Intestinal obstruction	CT: Distended loops of the small intestine.	—	SM	—	Incomplete resection	—	None	Death
7	20361382	2010^36)^	51	Male	Abdominal pain, fatigue	None	None	MRI: A tumor above the aortic bifurcation with encasement of the aorta and the vena cava	CT-guided biopsy was inconclusive	SM	—	Incomplete resection	9.7 × 7.7 × 5.9	Steroids, azathioprine	Sustained remission
8	20671922	2010^37)^	75	Male	Vague abdominal discomfort	None	None	CT: Heterogeneous, soft tissue, mesenteric mass with calcifications	Laparoscopic biopsy	SM	—	None	—	Steroids, azathioprine, tamoxifen	Sustained remission
9	22980600	2012^38)^	41	Male	Abdominal pain, bloating, diarrhea, tenesmus	None	None	CT: A thickened colon wall surrounded by hazy, striated mesenteric fat	Laparoscopic biopsy	SM	—	Colostomy (palliative surgery)	—	Steroids, tamoxifen, colchicine, aspirin	Sustained remission
10	25326572	2014^39)^	61	Female	Gastrooesophageal reflux, Af	AT, BSO, limited systemic sclerosis	None	CT: No significant metabolic activity. FDG-PET: Negative	CT-guided biopsy revealed FN	MP	—	None	—	D-penicillamine	Sustained remission
11	25434322	2014^40)^	7	Female	Abdominal pain, vomiting, diarrhea, fever	None	None	CT: Subtle attenuation in the mesentery	Surgical biopsy	SM	Elevated serum IgG4 level IgG4-positive plasma cells in IHC	None	—	Steroids, azathioprine, colchicine	Sustained remission
12	26629850	2015^41)^	80	Male	Abdominal pain	Dementia, Af, HT, HL, epilepsey	None	CT: Ill-defined increase in the density of the peritoneal fat	—	MP	—	None	—	Steroids	Sustained remission
13	30038982	2016^42)^	33	Female	Abdominal pain, Anorexia, Nausea	None	None	CT: A round, encapsulated mass. US: Oval hyperechogenic mass with a hypo-echogenic rim	—	Encapsulated Mesenteric FN	—	—	—	—	—
14	26781011	2016^43)^	70	Female	Abdominal pain, appetite loss, fever	Sjögren’s syndrome	None	CT: Increased concentration of mesenteric fat	Laparoscopic biopsy	MP	—	Ileostomy (palliative surgery)	—	Steroids	Sustained remission
15	27099861	2016^44)^	63	Male	Abdominal pain	Laparotomy for probable cyst on pancreas cholecystectomy	None	CT: Pseudocapsule, fat ring sign	Surgical biopsy	SM	Normal serum IgG4 level	None	—	Steroids, tamoxifen	Sustained remission; relapsed with follicular lymphoma at 5 years
16	27437192	2016^45)^	13	Male	Abdominal pain	None	None	CT: Increased attenuation and enhancement of the omental fat anterior peritoneal wall thickening and enhancement	Laparoscopic biopsy	MP	—	None	—	Steroids	Sustained remission
17	28270878	2017^46)^	77	Male	Progressive dyschezia, abdominal pain	HT, DM	None	CT: Thickened mesentery, enlarged lymph nodes, strand-like densities	—	SM	Not inspected	Complete resection	—	None	No recurrence
18	28638005	2017^47)^	80	Male	Abdominal pain, tenderness	HT, CKD with PD	Intestinal perforation	CT: Fat-ring sign, peritoneal calcifications	—	SM	IgG4 negative	None	—	None	Death
19	28764764	2017^48)^	5	Male	Recurrent bloating, abdominal pain, anorexia, vomiting	None	None	CT: Bowel wall thickening, massive ascites	—	SM	Not inspected	Incomplete resection	—	Steroids	No recurrence
20	29632756	2018^49)^	60	Male	Abdominal pain, distension, appetite loss	Abdominal stab wound and laparotomy	None	CT: Mesenteric mass with surrounding stranding and poorly defined borders	Surgical biopsy	SM	Normal serum IgG4 level POD2–3 IgG4-positive plasma cells in IHC	Incomplete resection	Approximately 8	None	No recurrence
21	32105017	2019^50)^	5 m	Female	Vomiting, abdominal distention, tenderness	None	Intestinal obstruction	CT: Widespread free fluids	Surgical biopsy	MP	No IgG4-positive lymphoplasmocytic infiltration	Not obvious	—	None	No recurrence
			10	Male	Abdominal pain, nausea, vomiting	None	Perforated appendicitis	US: Free fluids and an edematous appendix	Surgical biopsy	MP	Normal serum IgG4 level	Not obvious	—	None	No recurrence
			15	Male	Vomiting	Juvenile idiopathic arthritis	None	MRI: A mass surrounding perirenal tissue and ureters, leading to retroperitoneal fibrosis	Surgical biopsy	MP	Normal serum IgG4 level IgG4-negative in IHC	Not obvious	—	Steroids, azathioprin, methotrexate, anti-IL6	Sustained remission
22	33235771	2020^1)^	69	Male	None	None	None	CT: Encapsulated mass with no CE. MRI: High intensity on T1WI, isointense on T2WI	—	Encapsulated FN	—	Complete resection	4 × 4	None	No recurrence
23	33564540	2021^51)^	55	Female	Abdominal pain	Gastroesophageal reflux, Gastritis, Depression, Seizure Cholecystectomy, Appendectomy	None	CT: Nonspecific, lobulated soft-tissue lesion	Impossible to perform	SM	—	Complete resection	2.0 × 1.5 × 3.5	None	No recurrence
24	33628534	2021^52)^	49	Male	Abdominal pain, Diarrhea, Weight loss, Night sweats, Fever	HT, HL, PTSD, Depression	None	CT: Hazy infiltration with subcentimeter lymph nodes,	Laparoscopic biopsy	SM	—	None	—	Steroids, tamoxifen to adalimumab, pentoxifylline	Sustained remission
25	33791401	2021^6)^	42	Male	Abdominal pain	Antiphospholipid syndrome	None	CT: Heterogeneous, non-calcified, with lobulated margins, mostly isodense. MRI: Encapsulated mesenteric mass, isointense on T1WI, hypointense on T2WI, no restricted diffusion on DWI	—	Mesenteric FN	—	Complete resection	5 × 3.7	None	—
26	33814933	2021^53)^	63	Male	Abdominal pain, Weight loss	None	None	CT: Misty mesentery. US: A well-defined hyperechoic mass	US-guided biopsy	SM	—	None	4.1 × 11	Steroids, tamoxifen, aspirin	Sustained remission
27	34490340	2021^54)^	23	Male	Mesogastric pain, Fatigue, Appetite loss, Weight loss	None	None	CT: Pseudocapsule and locoregional lymph nodes of increased volume	—	SM	IgG4-negative in IHC	Complete resection	8.5 × 8 × 6.5	None	No recurrence
28	34836933	2021^55)^	83	Female	Abdominal pain, Vomiting	Right Inguinal hernia repair	Strangulated inguinal hernia	CT: A thickened intestinal wall in multiple loops in the context of a right inguinal hernia	—	SM	—	Complete resection	—	—	—
29	34970377	2021^11)^	60	Male	Abdominal pain	HT, DM	None	CT: Hypodense amorphous collection versus mass within mesentery	Nonspecific	SM	—	Complete resection	7.2 × 6.4 × 3.3	—	No recurrence
30	36185930	2022^56)^	69	Female	Abdominal pain, Nausea	Fibromyalgia	None	CT: Sub-centimeter lymph nodes and surrounding haziness and stranding in the root of the mesentery	FN	SM	—	None	—	—	Diagnosed with melanoma and lymphoma several years later
31	40149014	2025^57)^	68	Female	Abdominal pain	Left Inguinal hernia repair	Intestinal volvulus	CT: Mesenteric volvulus accompanied by intestinal obstruction	—	SM	—	Complete resection	4.0 × 3.5 × 3.0	None	No recurrence
32	(Our case)	2026	85	Male	None	Rectal cancer, Appendectomy, Oral cancer, DM and Hashimoto’s disease	None	CT: A mass in the root of the mesentery, no misty sign. MRI: A mesenteric mass with slightly higher signal intensity than muscle on T1WI, an isointense signal on T2WI, diffusion restriction on DWI. FDG-PET: Focal uptake on the mass	Impossible to perform	Mesenteric FN	Not inspected	Complete resection	2.1 × 2.0	None	No recurrence

Af, atrial fibrillation; AT, abdominal total hysterectomy; BSO, bilateral salpingo-oophorectomy; CKD, chronic kidney disease; DM, diabetes mellitus; DWI, diffusion-weighted imaging; FDG-PET, ^18^F-fluorodeoxyglucose PET; FN, fat necrosis; HL, hyper lipidemia; HT, hypertension; IHC, immunohistochemistry; MP, mesenteric panniculitis; PD, Parkinson’s disease; PTSD, post-traumatic stress disorder; RM, retractile mesenteritis; SM, sclerosing mesenteritis; T1WI, T1-weighted images; T2WI, T2-weighted images; US, ultrasonography

The selected reports are summarized in **Table 2**. The diagnostic difficulty reflected in these reports is consistent with the series reported by Akram et al., in which retrospective analysis of all 92 cases of sclerosing mesenteritis required tissue confirmation, with laparotomy performed in 65% of cases, laparoscopic biopsy in 25%, and CT-guided biopsy in 10%.^14)^ A literature review by Watanabe et al. identified only 9 abdominal cases of encapsulated fat necrosis through 2020.^1)^ Only a handful of mesenteric cases have been reported since then, including cases associated with systemic disease or mimicking malignant neoplasms.^2,6,12)^ Taken together, these cases indicate that the main clinical significance of mesenteric fat necrosis lies not only in its rarity but also in the difficulty of distinguishing it from malignant mesenteric tumors when it presents as a mass-forming lesion.

In retrospect, the possibility of IgG4-related disease associated with sclerosing mesenteritis should also have been considered. Recent expert guidance recommends considering serum IgG4 as part of the evaluation of suspected sclerosing mesenteritis.^7)^ At the same time, the relationship between sclerosing mesenteritis and IgG4-related disease remains incompletely defined, and not all cases of sclerosing mesenteritis fulfill the clinicopathologic criteria for IgG4-related disease.^58,59)^ In the present case, the serum IgG4 level was not measured at the time of diagnosis, and retrospective confirmation was not feasible. We have therefore acknowledged this as a limitation of the present report.

If a confident preoperative diagnosis of mesenteric fat necrosis had been established, careful observation might have been a reasonable management option, particularly in an asymptomatic patient such as ours. Although reports of mesenteric fat necrosis itself treated nonoperatively are extremely limited, some patients with sclerosing mesenteritis have been successfully managed with corticosteroids and have maintained remission without surgery. At the same time, many previously reported cases ultimately required laparoscopic or open biopsy, or diagnostic resection, because a definitive diagnosis could not be established preoperatively, suggesting how difficult preoperative diagnosis can be in practice.

## CONCLUSIONS

This report described an uncommon case of mesenteric fat necrosis that can closely mimic a malignant tumor and should be considered in the differential diagnosis of an enlarging mesenteric mass. When interval growth and suspicious multimodality imaging findings were present and biopsy was not feasible, surgical resection was pivotally important for diagnosis, underscoring the need to consider this entity in the differential diagnosis of mesenteric masses.
